# Relationships between *in vivo* surface and *ex vivo* electrical impedance myography measurements in three different neuromuscular disorder mouse models

**DOI:** 10.1371/journal.pone.0259071

**Published:** 2021-10-29

**Authors:** Sarbesh R. Pandeya, Janice A. Nagy, Daniela Riveros, Carson Semple, Rebecca S. Taylor, Benjamin Sanchez, Seward B. Rutkove

**Affiliations:** 1 Department of Neurology, Beth Israel Deaconess Medical Center, Harvard Medical School, Boston, MA, United States of America; 2 Department of Electrical and Computer Engineering, University of Utah, Salt Lake City, Utah, United States of America; Emory University, UNITED STATES

## Abstract

Electrical impedance myography (EIM) using surface techniques has shown promise as a means of diagnosing and tracking disorders affecting muscle and assessing treatment efficacy. However, the relationship between such surface-obtained impedance values and pure muscle impedance values has not been established. Here we studied three groups of diseased and wild-type (WT) animals, including a Duchenne muscular dystrophy model (the D2-mdx mouse), an amyotrophic lateral sclerosis (ALS) model (the SOD1 G93A mouse), and a model of fat-related atrophy (the db/db diabetic obese mouse), performing hind limb measurements using a standard surface array and *ex vivo* measurements on freshly excised gastrocnemius muscle. A total of 101 animals (23 D2-mdx, 43 ALS mice, 12 db/db mice, and corresponding 30 WT mice) were studied with EIM across a frequency range of 8 kHz to 1 MHz. For both D2-mdx and ALS models, moderate strength correlations (Spearman rho values generally ranging from 0.3–0.7, depending on the impedance parameter (i.e., resistance, reactance and phase) were obtained. In these groups of animals, there was an offset in frequency with impedance values obtained at higher surface frequencies correlating more strongly to impedance values obtained at lower *ex vivo* frequencies. For the db/db model, correlations were comparatively weaker and strongest at very high and very low frequencies. When combining impedance data from all three disease models together, moderate correlations persisted (with maximal Spearman rho values of 0.45). These data support that surface EIM data reflect *ex vivo* muscle tissue EIM values to a moderate degree across several different diseases, with the highest correlations occurring in the 10–200 kHz frequency range. Understanding these relationships will prove useful for future applications of the technique of EIM in the assessment of neuromuscular disorders.

## Introduction

Surface electrical impedance myography (EIM) is a non-invasive method for assessing muscle condition via the application of an alternating electrical current across a range of frequencies and measurement of the resulting voltages [1]. The technology is showing potential utility as a primary diagnostic of neuromuscular disorders but also as a means of assessing disease progression or response to therapy. Conditions ranging from amyotrophic lateral sclerosis (ALS) [2–4], Duchenne muscular dystrophy (DMD) [5–7], as well as less severe conditions, such as disuse atrophy [8, 9] or sarcopenia [10–13] can be detected and tracked by EIM.

To date in humans, most studies have been performed using surface techniques [1], although needle techniques have also been recently introduced [14–17]. In surface approaches, an electrode array is applied to a limb and measurements are made of the underlying tissue from which three primary measures are derived: resistance, reactance, and phase angle. Such measures reflect the passive electrical properties of the underlying muscle but are also impacted to some extent by other tissues underneath the electrodes such as skin and subcutaneous fat, as well as the size and geometry of the limb [18, 19]. The impact of these underlying tissues relies on a number of factors, including the thickness of the skin/subcutaneous fat layer and the volume of muscle tissue present.

The standard approach for obtaining a “ground truth” or reference measure of impedance values of tissue consists of measuring the intrinsic electrical properties of tissue (namely its conductivity and relative permittivity) [20]. One invasive method of obtaining such information consists of excising a small sample of muscle tissue, trimming it into a cube with known geometrical dimensions and then measuring its impedance in a dielectric cell, and thus providing a so-called *ex vivo* measurement [1]. While such *ex vivo* measurements can provide important insights into muscle condition, the impedance values and the derived intrinsic electrical properties (obtained after calibration by accounting for the contribution of the *ex vivo* cell itself to the recorded signal) are impacted by experimental conditions (e.g., postmortem time and temperature) and will naturally differ compared to data obtained *in vivo* [21, 22]. Nevertheless, there is an expectation that there should be consistent relationships between surface and *ex vivo* approaches.

In this study, we sought to understand the relationship between surface impedance values of muscle obtained *in vivo* and impedance values obtained *ex vivo* in an impedance-measuring cell in three different murine models of neuromuscular disease: the mouse ALS SOD1 G93A model [23], the D2-mdx model of Duchenne muscular dystrophy [24], and the diabetic obese db/db mouse model [25], in which muscle atrophy without primary muscle disease is present. We had three questions: 1. How well do surface impedance values correlate to *ex vivo* impedance data at our “standard” range of frequencies (approximately 30–300 kHz); 2. Is there a shift in the frequency correlations between and surface and *ex vivo* impedance values, such that, for example, impedance values obtained at higher surface frequencies correlate best to impedance values obtained at lower *ex vivo* frequencies; and 3. Are there differences in these relationships among these animal models?

## Methods

### Animals

All experimental procedures were approved by the Institutional Animal Care and Use Committee at Beth Israel Deaconess Medical Center. All animals were fed standard chow *ad libitum* and housed in standard fashion of groups up to 5 in microisolator cages in the Slosberg/Landay Animal Facility equipped with a 12:12 light/dark standard lighting cycle (lights on at 7:00AM; lights off at 7:00PM). All cages are checked daily by the animal care technical staff to confirm animal health, feed and water and cage condition. In addition, all rodent rooms use sentinel animals to monitor the health status of the room. Sentinel animals are tested for quarterly at Charles River Diagnostic Labs for a complete health screen to include bacteriology, pathology and parasitology. Experimental animals undergo regular weekly health and behavioral assessments (including body mass, motor score, mobility and gait, ability to feed, paw grip endurance, paw grip strength), and electrophysiological studies (electrical impedance myography). For the analyses completed here, impedance data acquired as part of three earlier studies [26–28] were utilized. The sample sizes for each group were thus those used in each of the earlier studies.

*1*. *Muscular dystrophy and controls*. Male wild type (WT) (DBA/2J; Strain #000671) and D2-mdx mice (D2.B10-*Dmd*^*mdx*^/J; Strain #013141) were obtained from Jackson Labs (Bar Harbor ME), and aged to 6, 13, 21, and 43 weeks. Animals were allowed to stabilize for 48 hr to recover from shipment prior to use in experimental protocols. There were a total of 23 D2-mdx and 17 WT mice.

*2*. *Obese mice and controls*. Male WT (C57BLKS/J; Strain #000662) and db/db mice (BKS.Cg-Dock7m +/+ Leprdb/J; Strain #000642) were obtained from Jackson Labs, (Bar Harbor ME), and aged to 6, 10, and 20 weeks in order to evaluate the impact of increasing fat deposition and skeletal muscle atrophy, both of which occur naturally as these animals age. Animals were allowed to stabilize for 48 hr to recover from shipment prior to use in experimental protocols. Mice (5 db/db and 5 WT) were evaluated at each age. Overall, the final analysis included 12 db/db and 13 WT mice.

*3*. *ALS mice and controls*. Breeding pairs of ALS (B6SJL-Tg(SOD1- G93A)1Gur/J) mice were obtained from Jackson Labs (Bar Harbor, ME) and bred to obtain 43 animals with ALS (approximately half female and half male). Animals were euthanized at various ages ranging from 8–18 weeks (approximately 6–7 animals per fortnight, at 8, 12, 14, 16, and 18 weeks). Of note, no WT animals were used here; however, the youngest of the animals at 8 weeks of age are generally presymptomatic. Mice with ALS develop symptoms and pathology resembling human ALS, with eventual paralysis in one or more hind limbs attributable to the loss of motor neurons from the spinal cord. Symptomatic ALS mice are monitored daily to assess feeding and movement. DietGel76A is provided when ALS animals can no longer access standard chow. The animals are checked twice per day when one of their hind limbs becomes paralyzed. When both hind limbs become paralyzed, the animal are euthanized. Euthanasia is performed by inhalation of carbon dioxide (CO_2_) gas delivered from a compressed gas canister.

#### EIM measurements

In all animal studies, after removing the fur with clippers, a depilatory agent was applied to the left hind limb to eliminate any remaining fur, and the skin was cleaned with 0.9% saline solution, as previously described [29]. The animal was placed in a prone position and both legs were taped to the measuring surface at an approximately 45° angle extending out from the body in preparation for measurements. A fixed rigid 4-electrode impedance-measuring surface array was applied over the left gastrocnemius muscle. EIM measurements were performed in an unblinded fashion with the mView system (Myolex, Inc, Boston, MA), which measures impedance at 41 frequencies from 1 kHz to 10 MHz as previously described [30, 31]. For this analysis, we removed data at frequencies above 1 MHz since these are prone to parasitic inductance and capacitance artifacts affecting the measurement, yielding EIM data for 29 frequencies. Data was collected with the array oriented such that electrical current was passed predominantly parallel (longitudinal) to the general myofiber direction.

After measurements were completed, the animals were euthanized and the gastrocnemius muscle excised and measured in an impedance measuring cell of 0.5 X 0.5 cm footprint (muscle height was generally approximately 0.4 cm) within approximately 10 minutes of the animal’s death. We used a Plexiglass dielectric measuring cell as described [20], with the fibers oriented perpendicularly to the metal plates (for longitudinal muscle measurements). Measuring the electrical properties of the excised muscle took approximately 3–5 minutes.

#### Data analysis

The statistical analyses of the impedance data were performed using GraphPad Prism V8 (GraphPad Software, Inc. La Jolla, CA) and R version 4.03. We used Spearman’s rank-order correlation to extract the correlation coefficient *(Rs)* between the various frequency metrics between these two measurement approaches (*Ex vivo* and Surface) across all animals, for the 3 major impedance values: resistance, reactance, and phase. We then used these values to create correlation heatmaps and reviewed individual frequency correlations to better understand the specific nature of this relationship.

## Results

The individual disease and cumulative correlation results for longitudinal phase, reactance and resistance are provided in Tables 1–3, respectively.

**Table 1:** Longitudinal phase (LP) value correlation.

Disease	Correlation	Spearman’s Rho Value (Rs)	p-value
D2-mdx with WT	50 kHz *Ex vivo* vs 50 kHz Surface	0.7	<0.001
	50 kHz *Ex vivo* vs 126 kHz Surface	0.76	<0.001
	15 kHz *Ex vivo* vs 50 kHz Surface	0.75	<0.001
	37 kHz *Ex vivo* vs 126 kHz Surface	0.78	<0.001
ALS	50 kHz *Ex vivo* vs 50 kHz Surface	0.49	0.002
	50 kHz *Ex vivo* vs 31 kHz Surface	0.53	0.001
	8 kHz *Ex vivo* vs 50 kHz Surface	0.61	<0.001
	8 kHz *Ex vivo* vs 31 kHz Surface	0.65	<0.001
db/db with WT	50 kHz *Ex vivo* vs 50 kHz Surface	0.22	0.293
	50 kHz *Ex vivo* vs 1027 kHz Surface	0.38	0.059
	862 kHz *Ex vivo* vs 50 kHz Surface	0.40	0.047
	428 kHz *Ex vivo* vs 607 kHz Surface	0.59	0.002
Overall with WT	50 kHz *Ex vivo* vs 50 kHz Surface	0.25	0.008
	50 kHz *Ex vivo* vs 150 kHz Surface	0.35	<0.001
	8 kHz *Ex vivo* vs 50 kHz Surface	0.41	<0.001
	9 kHz *Ex vivo* vs 89 kHz Surface	0.44	<0.001

^a^ Correlation coefficients (Spearman’s Rho) and p values for: 50 kHz *Ex vivo* and 50 kHz Surface impedance measurements, maximum correlation coefficient given *Ex vivo* impedance frequency is at 50 kHz, maximum correlation coefficient given Surface impedance frequency is at 50 kHz, and maximum correlation coefficient within the spectrum for the given datasets of diseases and its combination.

**Table 2:** Longitudinal reactance (LX) value correlations.

Disease	Correlation	Spearman’s Rho Value (Rs)	p-value
D2-mdx with WT	50 kHz *Ex vivo* vs 50 kHz Surface	0.48	0.002
	50 kHz *Ex vivo* vs 22 kHz Surface	0.55	<0.001
	15 kHz *Ex vivo* vs 50 kHz Surface	0.57	<0.001
	15 kHz *Ex vivo* vs 18 kHz Surface	0.66	<0.001
ALS	50 kHz *Ex vivo* vs 50 kHz Surface	0.3	0.077
	50 kHz *Ex vivo* vs 31 kHz Surface	0.31	0.07
	9 kHz *Ex vivo* vs 50 kHz Surface	0.34	0.053
	359 kHz *Ex vivo* vs 862 kHz Surface	0.51	0.002
db/db with WT	50 kHz *Ex vivo* vs 50 kHz Surface	0.5	0.011
	50 kHz *Ex vivo* vs 8 kHz Surface	0.67	<0.001
	15 kHz *Ex vivo* vs 50 kHz Surface	0.7	<0.001
	13 kHz *Ex vivo* vs 8 kHz Surface	0.76	<0.001
Overall with WT	50 kHz *Ex vivo* vs 50 kHz Surface	0.29	0.002
	50 kHz *Ex vivo* vs 178 kHz Surface	0.41	<0.001
	9 kHz *Ex vivo* vs 50 kHz Surface	0.37	<0.001
	11 kHz *Ex vivo* vs 178 kHz Surface	0.46	<0.001

^a^ Correlation coefficients (Spearman’s Rho) and p values for: 50 kHz *Ex vivo* and 50 kHz Surface impedance measurements, maximum correlation coefficient given *Ex vivo* impedance frequency is at 50 kHz, maximum correlation coefficient given Surface impedance frequency is at 50 kHz, and maximum correlation coefficient within the spectrum for the given datasets of diseases and its combination.

**Table 3:** Longitudinal Resistance (LR) value correlations.

Disease	Correlation	Spearman’s Rho Value (Rs)	p-value
D2-mdx with WT	50 kHz *Ex vivo* vs 50 kHz Surface	0.32	0.043
	50 kHz *Ex vivo* vs 1027 kHz Surface	0.46	0.003
	13 kHz *Ex vivo* vs 50 kHz Surface	0.44	0.005
	862 kHz *Ex vivo* vs 1027 kHz Surface	0.53	<0.001
ALS	50 kHz *Ex vivo* vs 50 kHz Surface	0.40	0.015
	50 kHz *Ex vivo* vs 89 kHz Surface	0.42	0.012
	50 kHz *Ex vivo* vs 50 kHz Surface	0.40	0.015
	50 kHz *Ex vivo* vs 89 kHz Surface	0.42	0.012
db/db with WT	50 kHz *Ex vivo* vs 50 kHz Surface	0.65	<0.001
	50 kHz *Ex vivo* vs 74 kHz Surface	0.66	<0.001
	150 kHz *Ex vivo* vs 50 kHz Surface	0.71	<0.001
	302 kHz *Ex vivo* vs 724 kHz Surface	0.73	<0.001
Overall with WT	50 kHz *Ex vivo* vs 50 kHz Surface	0.47	<0.001
	50 kHz *Ex vivo* vs 253 kHz Surface	0.49	<0.001
	9 kHz *Ex vivo* vs 50 kHz Surface	0.51	<0.001
	9 kHz *Ex vivo* vs 62 kHz Surface	0.52	<0.001

^a^ Correlation coefficients (Spearman’s Rho) and p values for: 50 kHz *Ex vivo* and 50 kHz Surface impedance measurements, maximum correlation coefficient given *Ex vivo* impedance frequency is at 50 kHz, maximum correlation coefficient given Surface impedance frequency is at 50 kHz, and maximum correlation coefficient within the spectrum for the given datasets of diseases and its combination.

**Fig 1:**
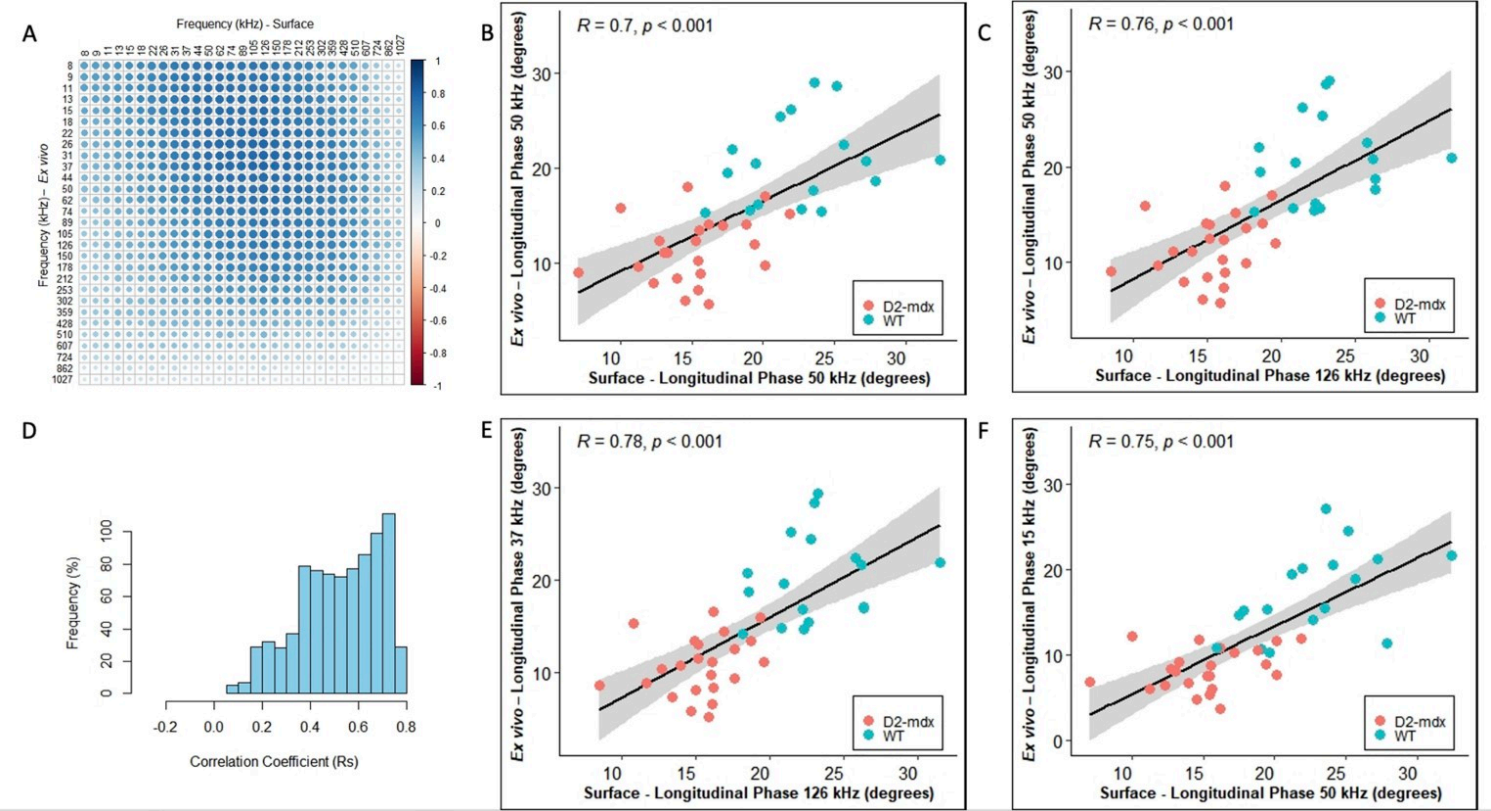
**D2-mdx with wild type (WT) mice data (left GA muscles) for longitudinal phase orientation (N = 40, 23 D2-mdx and 17 WT = DBA/2J). A.** Heat-map with correlation coefficients related to individual frequency spectrum on *Ex vivo* and Surface impedance (phase) measurements, **B.** Linear correlation plot for 50 kHz *Ex vivo* and 50 kHz Surface impedance (phase) measurements, **C.** Linear correlation plot of surface vs *ex vivo* phase values at frequencies corresponding to the maximum correlation coefficient given *Ex vivo* impedance (phase) frequency is at 50 kHz, **D.** Histogram indicating the frequency of all possible correlation coefficients (Rs), **E.** Linear correlation plot of surface vs *ex vivo* phase values at frequencies corresponding to the maximum correlation coefficient within the spectrum and **F.** Linear correlation plot of surface vs *ex vivo* phase values at frequencies corresponding to the maximum correlation coefficient given Surface impedance (phase) frequency is at 50 kHz.

**Fig 2:**
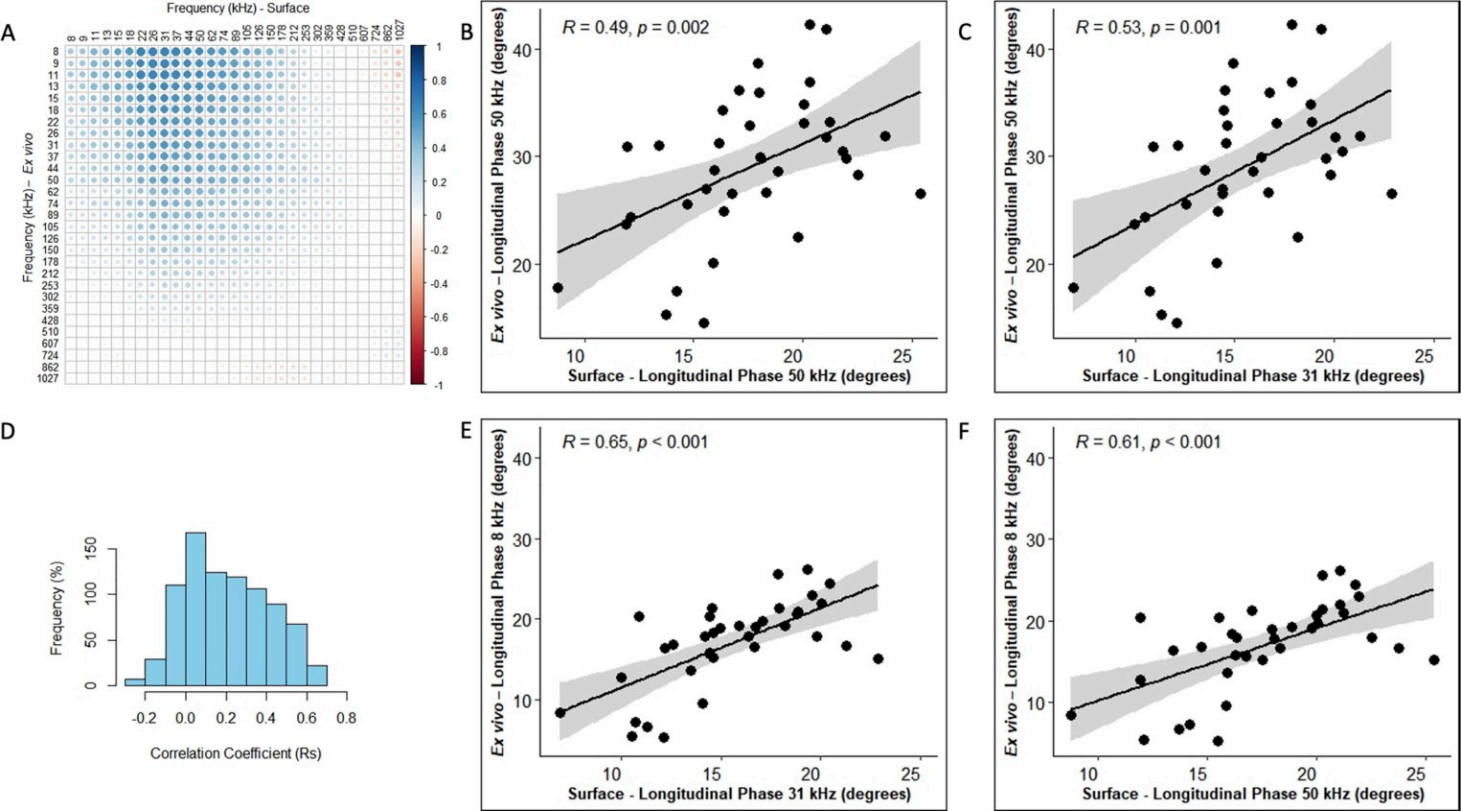
**ALS mice data (left GA muscles) for longitudinal phase orientation (N = 36). A.** Heat-map with correlation coefficients related to individual frequency spectrum on *Ex vivo* and Surface impedance (phase measurements, **B.** Linear correlation plot for 50 kHz *Ex vivo* and 50 kHz Surface impedance (phase) measurements, **C.** Linear correlation plot of surface vs *ex vivo* phase values at frequencies corresponding to the maximum correlation coefficient given *Ex vivo* impedance (phase) frequency is at 50 kHz, **D.** Histogram indicating the frequency of all possible correlation coefficients (Rs), **E.** Linear correlation plot of surface vs *ex vivo* phase values at frequencies corresponding to the maximum correlation coefficient within the spectrum and **F.** Linear correlation plot of surface vs *ex vivo* phase values at frequencies corresponding to the maximum correlation coefficient given Surface impedance (phase) frequency is at 50 kHz.

**Fig 3:**
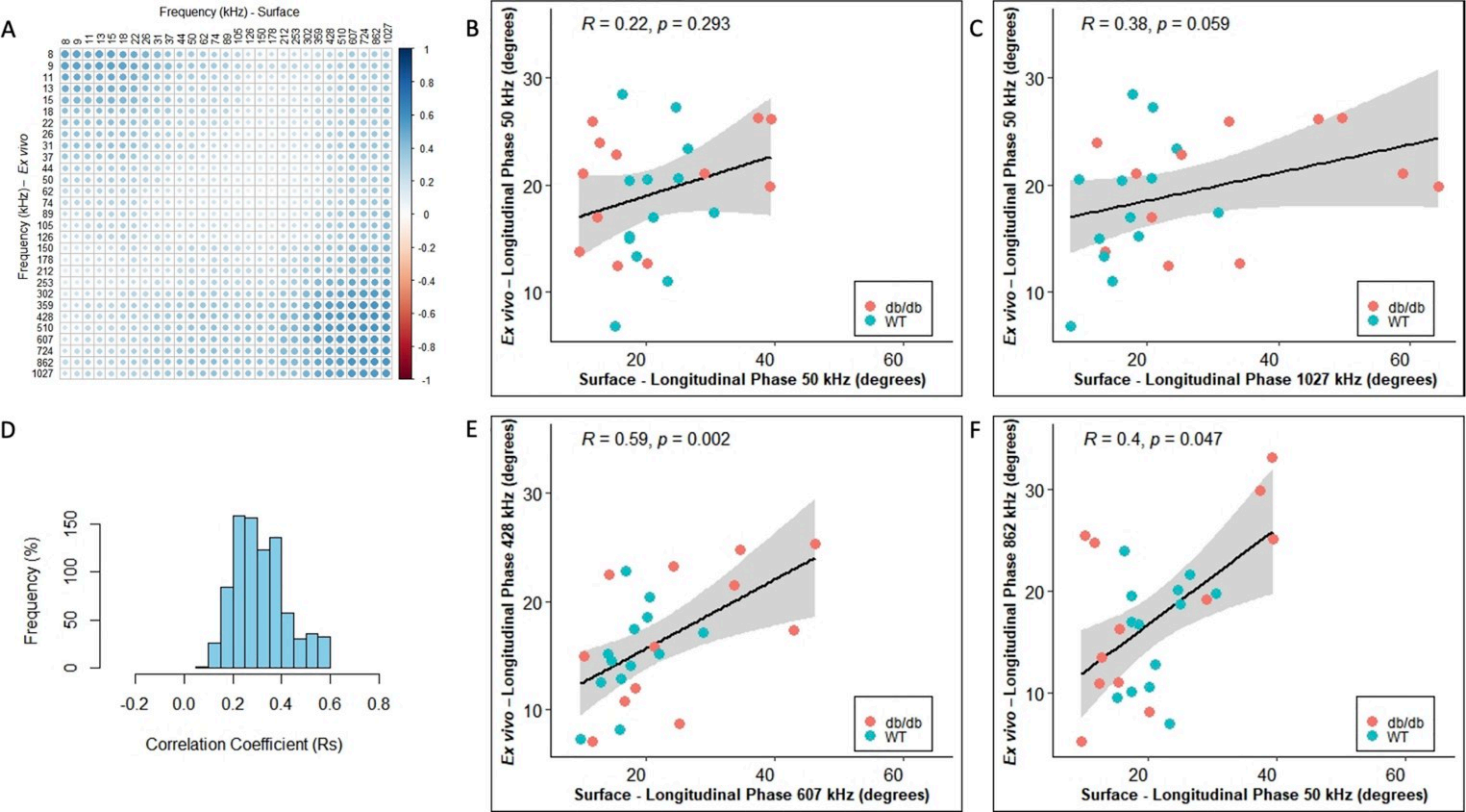
**db/db with Wild Type (WT) mice data (left GA muscles) for longitudinal phase orientation (N = 25, 12 db/db and 13 WT = C57Bl/6). A.** Heat-map with correlation coefficient related to individual frequency spectrum on *Ex vivo* and Surface impedance (phase) measurements, **B.** Linear correlation plot for 50 kHz *Ex vivo* and 50 kHz Surface impedance (phase) measurements, **C.** Linear correlation plot of surface vs *ex vivo* phase values at frequencies corresponding to the maximum correlation coefficient given *Ex vivo* impedance (phase) frequency is at 50 kHz, **D**. Histogram indicating the frequency of all possible correlation coefficients (Rs), **E.** Linear correlation plot of surface vs *ex vivo* phase values at frequencies corresponding to the maximum correlation coefficient within the spectrum and **F.** Linear correlation plot of surface vs *ex vivo* phase values at frequencies corresponding to the maximum correlation coefficient given Surface impedance (phase) frequency is at 50 kHz.

**Fig 4:**
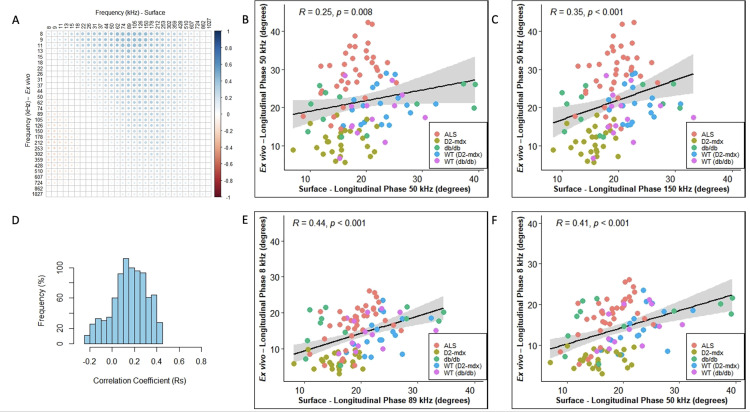
Combination of all given mice data (N = 111). **A.** Heat-map with correlation coefficient related to individual frequency spectrum on *Ex vivo* and Surface impedance (phase) measurements, **B.** Linear correlation plot for 50 kHz *Ex vivo* and 50 kHz Surface impedance (phase) measurements, **C.** Linear correlation plot of surface vs *ex vivo* phase values at frequencies corresponding to the maximum correlation coefficient given *Ex vivo* impedance (phase frequency is at 50 kHz, **D.** Histogram indicating the frequency of all possible correlation coefficients (Rs), **E.** Linear correlation plot of surface vs *ex vivo* phase values at frequencies corresponding to the maximum correlation coefficient within the spectrum and **F**. Linear correlation plot of surface vs *ex vivo* phase values at frequencies corresponding to the maximum correlation coefficient given Surface impedance (phase) frequency is at 50 kHz.

In all cases, we included healthy animals (in the case of the ALS, the youngest animals i.e., 8 and 10 weeks which are typically presymptomatic). Since phase tends to be the preferred impedance measure for many studies, we then created frequency correlation matrices for the phase values in the form of heatmaps of the correlation coefficient (Rs) for each of these disease models (Panel A in Figs 1–4). The abscissa (x-axis) shows the surface frequencies and the ordinate (y-axis) shows the *ex vivo* frequencies. In addition to the correlation matrices with the intensity of blue shading mirroring the intensity of the correlation, we also provide a histogram of Spearman rho values (Rs) to provide a quantitative sense (i.e. frequency %) of the strength of the correlations (Panel D in Figs 1–4). Finally (in Figs 1–4), we provide a series of 4 scatterplots showing the relationships between the phase values obtained at: 50 kHz for both surface and *ex vivo* (Panel B); the surface frequency with the highest Rs value with 50 kHz *ex vivo* (Panel C); the 50 kHz surface frequency with the *ex vivo* frequency with the highest Rs values (Panel F); and finally, the correlation with the highest Rs value from the entire frequency spectrum (Panel E).

The strongest correlations between surface and *ex vivo* impedance data were found for the D2-mdx animals with the Rs values in the 0.32–0.78 range, and many with p<0.001. The ALS animals had the second highest range of Rs values (ranging from 0.30 to 0.65), with phase and resistance showing the strongest correlations and reactance somewhat weaker. The db/db animals showed the widest range of Rs values ranging from 0.22 to 0.76, with overall the weakest significance levels. It is important to note, however, that the p values are not easily compared across groups since the number of animals evaluated with each disorder and their respective WT were not identical. When grouping all the animals together, the highest Rs values were 0.46, 0.46, and 0.52, for phase, reactance, and resistance, as show in Tables 1, 2 and 3.

In terms of frequency-to-frequency correlation, for D2-mdx/WT animals, impedance values at slightly higher surface frequencies corresponded to impedance values at lower *ex vivo* frequencies. Similarly, in ALS animals, a similar trend was present, although in a much more restricted set of frequencies. This can be easily observed in Figs 1A and 2A and with the most intense blue region being shifted to the upper right. The data in the case of the db/db animals was far less consistent, showing the best correlations at the extremes of the frequency range (low frequency *ex vivo* values correlating to low frequency surface values, and high frequency *ex vivo* values corresponding to high frequency surface values (Fig 3A). When combining all the data sets together in Fig 4A, the same shift is apparent with impedance at higher surface frequencies generally correlating better to impedance at lower *ex vivo* frequencies with the 50 kHz phase surface value correlating most strongly to the 8 kHz *ex vivo* phase value.

## Discussion

Despite years of surface and *ex vivo* EIM data collection in mice and rats, this study represents the first effort to relate surface impedance values directly to *ex vivo* data. The results presented here confirm, as expected, that there is an association between both sets of measurements, but that this relationship, at the frequencies of interest (e.g., 50–100 kHz), is of only moderate strength. There are likely a number of reasons for this incongruity. First, there is the fact that *ex vivo* and surface EIM are measuring different tissues, since *ex vivo* measurements are performed directly on the excised muscle and there are no other intervening tissues. By contrast, surface measurements are impacted by the presence of skin and subcutaneous fat overlying the muscle. Second, the volumes of tissue being measured are different given the varying size and shape of the animal limb, whereas the *ex vivo* impedance measurements are restricted entirely to the block of tissue utilized in the impedance cell. Third, surface data is more likely to be impacted by contact artifacts at the interface of the array with the skin (this issue tends to be more problematic in mouse studies than in human given the very small size of the array relative to the size of the particular muscle under investigation). Fourth, given the anisotropic nature of muscle, with myofibers being extended cylindrical structures, the surface longitudinal measurements obtained are not truly longitudinal. This is in contrast to the *ex vivo* measurements in which there is an effort to ensure that the myofibers are aligned precisely with the metal plates which serve as the current electrodes. Finally, there are simply different experimental errors in both types of measurements, as the exact direction of muscle fibers is unknown and it is difficult to align the surface array with the muscle fibers or the perfectly trim the excised piece of muscle into a cube.

How do these factors relate to human EIM studies? In general, we would expect similar findings. Although human skin and subcutaneous fat thickness taken together are much greater than those of mice, it is helpful to interpret this difference in relation to the inter-electrode distances of the electrode array. The farther apart are the current electrodes, the greater the muscle penetration of the electrical current will be [19]. Our mouse array has inter-electrode distances of 3.5 mm [30] between the current and voltage electrodes, and mouse skin and subcutaneous fat thickness is approximately 1–2 mm, giving a ratio of approximately 3.5–1.75 (interelectrode distance: fat thickness). A human commercial array has a 2.54 cm current-voltage interelectrode distance [3], and human fat thickness can vary widely, from just 3 mm (in the anterior forearms of a thin individual) to 5 or more cm (in the abdomen of a moderately obese individual). This could give ratios of >7.5 (for some with little fat) to as low as 0.5 (for an obese individual). However, in a typical muscle with typical subcutaneous fat thickness, the ratio is likely to be similar to those of mice. Thus, based on this simple analysis, we would anticipate that these relationships between surface and *ex vivo* impedance data should generally hold for most appendicular muscles in non-obese individuals. However, good electrical contact on the skin is generally easier to achieve in humans and thus could even lead to an improved relationship in humans between surface and *ex vivo* measurements.

One interesting observation was that in longitudinal impedance measurements, there seem to be a slight shift with surface impedance values at higher frequencies correlating better to *ex vivo* impedance values at lower frequencies in both the ALS and D2-mdx models, as well as when data from all disease models was combined, but not in the db/db and corresponding WT animals alone. This likely makes conceptual sense since skin and fat tend to be more reactive (at high frequencies) [31] and thus their presence when performing surface EIM could cause this shift towards higher frequencies in the surface measurements.

It remains challenging to explain the differences among the heat maps in the different disorders studied here. Whereas the ALS and D2-dmx mouse correlations appear reasonably similar, the db/db model appears quite different. Much of this difference may be related to the fact that the surface electrode array in this condition is being impacted more greatly by the abundance of subcutaneous fat. If that is the case, the highest correlations would presumably connect subcutaneous fat with intramuscular fat. Indeed, the best correlation was found between two high frequencies for phase (ex vivo 428 kHz vs. surface 607 kHz). Another unclear aspect is why reactance values seemed to correlate better than phase values for only the db/db mice. Again, our suspicion is that this relates to the abundance of subcutaneous fat in this model, but we cannot hypothesize beyond that one concept.

One limitation of this analysis worth highlighting is that the *ex vivo* data also does not represent anything approaching a gold standard. This is in part due to the difficulty of manipulating the gastrocnemius muscle from mice. Trimming excised muscle from the animal into a perfect cube with dimensions 0.5 x 0.5 x 0.5 cm^3^ is technically challenging. Moreover, it is technically challenging to insert the excised muscle correctly into the impedance measuring cell, and ensure that the muscle slab is oriented with the fibers perpendicular to the current plate electrodes. In practice, there will be some mix of longitudinal and transverse data present. Finally, the tissue itself is no longer alive by the time it is placed in the impedance cell and thus the absence of blood flow and reduction in temperature can impact the data.

Another important observation from our analysis is that we observed minimal negative sensitivity in our studies. While this is an abstract concept, impedance measurement theory shows that impedance values can sometimes increase or decrease in counterintuitive directions based on the directionality of current flow, the electrodes’ characteristics and the tissues’ electrical properties within that region [18]. Thus, it remained conceivable that we could identify frequency ranges where strong negative correlations were present (e.g., increasing surface reactance could correspond to decreasing *ex vivo* reactance). The virtual absence of any strong negative correlations in this analysis also helps alleviate a concern that could prove very challenging for the technique’s clinical application if that were true.

It is important to realize, however, that the impedance values of phase, resistance, and reactance are not absolutes and do not reflect the actual condition of the tissue as resistance and reactance values are entirely influenced by the distance between the electrodes and the size of the electrodes [32]. While these effects are counterbalanced to some extent in the calculation of the phase value (phase = arctan(X/R)), they will still influence that result as well. The material properties, namely the conductivity and relative permittivity, represent the standard absolute of the muscle tissue [20]. We have not attempted to correlate surface EIM measurements to those intrinsic values, but the fact that the surface EIM data correlates to *ex vivo* EIM data to moderate extent at certain frequencies suggests that a similar relationship will hold between surface values and those intrinsic material properties.

We have previously shown that surface and *ex vivo* EIM data both correlate to histological features of muscle in a variety of conditions [26, 27, 33–35], so have not repeated that effort here. Rather, our goal was simply to establish what our expectations should be in processing surface impedance data in relation to *ex vivo* values and to consider the possibility that the surface frequencies of interest more strongly relate to lower frequency *ex vivo* values, at least in the D2-mdx and ALS animals.

We also note that there is a middle ground between *ex vivo* and surface measurements, namely measurement using indwelling needle electrodes. Needle electrodes could theoretically be placed subcutaneously or intramuscularly, but, to date, most of the work completed using needle electrode arrays has been performed intramuscularly [15, 33, 36, 37]. In fact, placing needles subcutaneously is quite challenging, and even small variations in position could greatly impact the impedance data obtained. Intramuscular electrodes, however, have the advantage of being completely surrounded by muscle and providing a truer measure of the impedance characteristics of the muscle itself. These studies can be performed using a multi-needle array or a single needle with multiple impedance electrodes embedded in it, and the latter is the subject of ongoing developmental and research efforts [38].

There are two important practical outcomes from this work. First, it helps confirm, perhaps unsurprisingly, that surface measurements in mice are capturing muscle condition and are not simply measuring volumetric or non-muscle tissues such as skin or subcutaneous fat, except perhaps in the db/db situation, where there is a markedly expanded subcutaneous volume. Second, it suggests that the best frequencies of interest to represent the condition of the underlying muscle using surface EIM may actually need to be somewhat different than those that have been typically chosen (e.g., surface phase values at 50 kHz) if one is attempting to correlate surface and *ex vivo* impedance values. It also, however, underscores the need to continue to refine EIM analytics so as to more effectively capture the muscle itself. Indeed, it may be that every disorder will have its optimal single surface frequency for tracking disease. But more to the point, the complexity of the relationship between surface and *ex vivo* values underscores the need to attempt to utilize the entire multifrequency spectrum, rather than a single frequency, whether comparing different disease states or tracking one individual over time as a disease progresses or remits. We have already explored the mathematical approaches for doing so [39], and using machine learning methodologies may also be possible. Finally, further studies can be pursued in humans, by performing surface measurements in conjunction with direct muscle measurements with a needle electrode [18] so that the underlying practice of surface EIM techniques can be optimized specifically for human use.
